# Chemical Biomarkers of Human Breast Milk Pollution

**DOI:** 10.4137/bmi.s564

**Published:** 2008-03-28

**Authors:** Francesco Massart, Giulia Gherarducci, Benedetta Marchi, Giuseppe Saggese

**Affiliations:** 1 Department of Pediatrics, Santa Chiara University Hospital of Pisa (Italy)

**Keywords:** breastfeeding, chemical contaminats, breast milk, environmental chemicals

## Abstract

Human milk is, without question, the best source of nutrition for infants containing the optimal balance of fats, carbohydrates and proteins for developing babies. Breastfeeding provides a range of benefits for growth, immunity and development building a powerful bond between mother and her child. Recognition of the manifold benefits of breast milk has led to the adoption of breast-feeding policies by numerous health and professional organizations such as the World Health Organization and American Academy of Pediatrics.

In industrially developed as well as in developing nations, human milk contamination by toxic chemicals such as heavy metals, dioxins and organohalogen compounds, however, is widespread and is the consequence of decades of inadequately controlled pollution. Through breastfeeding, the mother may transfer to the suckling infant potentially toxic chemicals to which the mother has previously been exposed.

In the present review, environmental exposure, acquisition and current levels of old and emerging classes of breast milk pollutants are systematically presented. Although scientific evidences indicated that the advantages of breast-feeding outweigh any risks from contaminants, it is important to identify contaminant trends, to locate disproportionately exposed populations, and to take public health measures to improve chemical BM pollution as possible.

## Introduction

Breastfeeding has been recognized and promoted by public health officials as the most beneficial source of nourishment during infancy (AAP, 1997; WHO, 2002). For example, a target goal of U.S. Department of Health and Human Services (HHS), was to promote early postpartum breastfeeding rates of 75% by the year 2010 (HHS, 2000). This encouragement on breastfeeding is motivated by well-known health effects of breast milk (BM). Epidemiological research showed that BM and breastfeeding of infants provide advantages with regard to general health, growth, and development while significantly decreasing risk for a large number of acute and chronic diseases (AAP, 1997; HHS, 2000; WHO, 2002).

However, potential risks associated with breastfeeding also need to be factored into the overall public health assessment when women are encouraged to breastfeed their newborn infants. Breastfeeding for nursing infants, can be a potential source of exposure to toxic chemicals to which the mother has previously been exposed (Solomon and Weiss, 2002). This may be true for two major reasons. First; breastfeeding serves as a food source for this segment of the human population: the diets of many newborns are limited to BM or it is at least an major nutrient source for suckling infants. Second; breastfed infants are at the top of the food chain. Therefore, chemicals accumulated in the mother’s tissues, may be transferred to infant during breastfeeding (Massart et al. 2005). This is especially true for environmental lipid-soluble pollutants such as polyhalogenated chemicals, because these chemicals tend to slowly degrade in the environment, to bioaccumulate and to bioconcentrate in the food chain, having long half-lives in humans (Massart and Meucci, 2007). Because of the milk fat content is relatively high, breastfeeding potentially causes high-dose exposure of lipid-soluble pollutants (Massart et al. 2005).

From the earliest reports in 1950s (Laug et al. 1951), over the years, many more chemicals have been assayed in human milk by improving analytic methods. The objective of the present study is to review available BM data focusing on both the old well-known and emerging classes of BM chemical contaminants as well as their environmental origins. It is of public health interest that the unquestionable benefits of breast-feeding regarding nutrition and psychological bonding should be advocated, but also that potentially hazardous substances in human milk be kept to a minimum to preserve infant health.

## Organochlorine Cyclodienes

Many organochlorine pesticide and insecticides detected in the BM studies chemically belong to the organochlorine cyclodiene family. Some examples are below.

### Dieldrin and aldrin

Dieldrin, along with aldrin, were popular croup pesticides till 1970s. Also, these chlorinated cyclodiene insecticides are used as prophylactic and treatment of timber against insect infestation caused by ants, termites and anobiid borers. Aldrin breaks down into dieldrin both in the body and in the environment. Once present in soil or water, dieldrin breaks down slowly, does not easily evaporate into the air, and binds to soil particles. Plants take up aldrin and dieldrin residues directly from the soil. In animals, including humans, dieldrin is stored in the fat and leaves the body very slowly (ATSDR, 1993). As of 1995, aldrin and dieldrin had been widely banned or severely restricted in more than 70 countries (PANNA, 1995).

Studies evaluating BM residues of aldrin and dieldrin have been conducted in at least 28 countries detecting in more than 99% BM samples in most of countries (WHO, 1989). Because of dieldrin is lipophilic and high lipid-concentration of human milk, BM level of dieldrin was generally 6-fold higher than blood level (WHO, 1989).

As countries restricted and banned both their chemical uses, through detection prevalence remained high, BM levels of dieldrin have significantly dropped till 10-fold decrease in the years following restriction (Noren and Meironyte, 2000; Jensen and Slorach, 1991; Furst et al. 1994; Currie et al. 1979; Shen et al. 2007; Khanjani and Sim, 2006). Conversely, in countries such as Kenya or U.S. Southern where dieldrin was heavily used, BM levels were often significantly higher (Rogan et al. 1980; Kanja et al. 1986).

### Chlordane

Chlordane, a mixture of more than 26 compounds, is the typical organochlorine cyclodiene pesticide. Chlordane has been used as agriculture pesticide, on home lawns and gardens and against termites. Chlordane has been banned in at least 47 countries and severely restricted in additional 14 (PANNA, 1995). Like most persistent organic pollutants, chlordane breakdown products have been detected in soil and also in fish, bird and mammal tissues up to 20 years after initial treatment (Jensen and Slorach, 1991; Savage et al. 1981).

BM studies examining chlordane metabolites have been conducted in at least 16 countries (Noren and Meironyte, 2000; Jensen and Slorach, 1991; Savage et al. 1981; Wickstrom et al. 1983; Taguchi and Yakushiji, 1988; Noren, 1993; Sudaryanto et al. 2006; Khanjani and Sim, 2006). Data from areas where chlordane was used, showed significant worldwide variability of BM levels associated with use patterns. For example, in the 1970s in the U.S., lactating women in the southern states had an average of 113 ng/g lipid (range 108–118 ng/g) of chlordane in their milk compared to 79 ng/g (range 76–82 ng/g) among women living in other regions, probably due to agricultural use and more intensive home termite control (Savage et al. 1981). Similarly, in Japan in the 1980s, women living in homes where chlordane was used for termite control, had BM levels 4.4-fold higher than women whose households used no form of chlordane (Taguchi and Yakushiji, 1988). While low BM contamination was reported in Indonesia and in Australia, slightly increasing levels of chlordane compounds were recently detected in Japanese primiparae suggesting that chlordane levels in Japanese BM have not decreased since 1998 (Sudaryanto et al. 2006; Khanjani and Sim, 2006; Kunisue et al. 2006).

Chlordane residues, however, have not been confined to regions where the chemical was used. For example, chlordane was detected in BM of Finland women in the mid-1980s, even though the chemical was never used in Finland and was heavily restricted in neighboring countries (Wickstrom et al. 1983). Exposure has been attributed to bioaccumulation in Baltic fish (Wickstrom et al. 1983). Despite the environmental persistence of chlordane, data from Sweden demonstrate a declining trend in average BM residues of chlordane metabolites in the decades since the chemical bans in most European countries (Noren and Meironyte, 2000; Noren, 1993). The peak concentrations of chlordane reported in the 1970s in Sweden were 4- to 5-fold lower than the contemporaneous U.S. concentrations (Jensen and Slorach, 1991).

### Heptachlor

Heptachlor is another organochlorine cyclodiene pesticide that has been used to control termites and as insecticide on seed grains and food crops. Heptachlor epoxide, the main metabolite of heptachlor, is extremely persistent detecting 14–16 years after application (WHO, 1988). Plants can draw heptachlor epoxide directly from the soil, and the chemical bioaccumulates in animals. Heptachlor has been banned or restricted in more than 60 countries (PANNA, 1995; WHO, 1988). However, some of these countries still permit its use for termite and other pest control, and many developing nations still use heptachlor for agricultural purposes (Noronha, 1998). Despite the U.S. ban on use in 1988, U.S. customs data showed that heptachlor was exported in large quantities through 1994 (PANNA, 1997). As countries have restricted and banned heptachlor, levels detected in BM have dropped, often by more than 10-fold (Jensen and Slorach, 1991; Currie et al. 1979; Chao et al. 2006).

### Hexachlorobenzene

Hexachlorobenzene (HCB) is a persistent organochlorine chemical that is both a seed fungicide and an industrial by-product in chlorination processes (e.g. manufacturing of wood preservatives, solvents and pesticides such as wastewater treatment) (Jensen and Slorach, 1991). HCB binds strongly to soil particles as well as to sediment and builds up in plants when it is present in soil (ATSDR, 1997).

Studies evaluating HCB in BM have been conducted in at least 34 countries (Solomon and Weiss, 2002). Historically, women in areas with less industrialization had significantly lower HCB levels in their BM. For instance, average HCB levels detected in BM in Kenya in the mid-1980s were just 1% of average levels found in Sweden and Germany at a similar time (Kanja et al. 1986). HCB levels in BM have declined in some industrialized countries over the past two decades, probably as a result of changes in fungicide use and procedural improvements in industry that have reduced HCB by-production (Noren and Meironyte, 2000; Jensen and Slorach, 1991). Indeed, higher BM levels of HCB were found in industrial areas of Czechoslovakia (Schoula et al. 1996). Furthermore, HCB levels in women living in the Kola Peninsula of Russia were twice as high as in Norway and in the Netherlands (Polder et al. 1998). Other Russian areas such as in Spain, have recently reported much higher levels of industrial pollution than other European areas (Tsydenova et al. 2007; Ribas-Fito et al. 2005). Similar to Sweden (Noren and Meironyte, 2000), Norway experienced a similar decrease, with BM levels of HCB dropping by 65% between the mid-1970s and early 1990s (Johansen et al. 1994). In opposite to Japanese data (Kunisue et al. 2006), also studies conducted in Germany, Belgium, Canada, Denmark, the Netherlands and Switzerland suggested a decline in HCB levels, with a decline of more than 85% in Germany (Jensen and Slorach, 1991).

### Hexachlorocyclohexane

Hexachlorocyclohexane (HCH) is an eight isomer mix insecticide. Different isomer forms have different levels of persistence and bioaccumulate in BM. The HCH γ-isomer, also known as lindane, is widely used as insecticide directly applied to the body and scalp to treat head and body lice. HCH is banned or severely restricted in more than 60 countries and lindane is specifically banned or restricted in 46 countries (PANNA, 1995), but its use is often permitted for special uses by exemption.

Studies evaluating HCH contamination of BM have been conducted in at least 43 countries (Jensen and Slorach, 1991; Subramanian et al. 2007; Sudaryanto et al. 2006; Jaraczewska et al. 2006; Chao et al. 2006; Cerna et al. 2007; Tsydenova et al. 2007). In general, countries that have monitored BM for HCH residues over time have witnessed a steady decrease. Clear downward trends in BM have been reported in Germany, Sweden and Japan (Noren and Meironyte, 2000; Jensen and Slorach, 1991; Furst et al. 1994; Yakushiji et al. 1979; Wilhelm et al. 2007; Kunisue et al. 2006; Shen et al. 2007). BM levels of HCH were extremely variable and often reflected differences in regional use and exposure patterns. In China and Japan, HCH was commonly used as an insecticide in rice fields, and levels as high as 6,500 ng/g of HCH in lipid have been measured in these countries (Jensen and Slorach, 1991). High BM levels of HCH were also reported in other Asian populations (Subramanian et al. 2007; Sudaryanto et al. 2006). Interestingly, a 1982 study in Norway, a decade after HCH was banned in that country, found higher β-HCH levels in women who had immigrated from developing countries (average level of 433 ng/g lipid) than native Norwegian women (about 80 ng/g lipid) (Skaare et al. 1988). The difference was attributed to the likelihood of higher exposures in developing countries.

Interestingly, in the absence of poisoning incidents or local exposure, dietary exposure has been shown to be an important predictor of BM levels of many permanent organic pollutants. In Germany, women who ate a healthy diet (low meat consumption and high vegetable and fruit intake) for at least 3 years had much lower HCH levels in their BM compared to women who ate more than 700 g of meat per week (Schade and Heinzow, 1998).

## Dioxins and Furans

Dioxins and furans are two closely related groups of chemical by-products that are produced throughout the world. Dioxins and furans are listed by several governmental and international agencies as known causes of human cancers hormone/reproductive disruptions, fetal abnormalities and immune alterations (EPA, 2002; Massart et al. 2006a).

Because dioxins and furans are environmentally persistent, lactation is one of the main routes of excretion (Massart et al. 2005). The dioxin and furan congeners thought to be most toxic to humans are 7 dioxins and 10 furans known as the 2,3,7,8-congeners. In BM monitoring studies, the term “dioxin” refers to this group of 17 congeners (Solomon and Weiss, 2002). Unlike other contaminants, there has been considerable work showing the effects of dioxin exposure at low levels near the range detected in BM (IARC, 1997).

Dioxins and furans have been BM-measured in at least 35 countries (Solomon and Weiss, 2002; IARC, 1997). It has been well established in the literature that dioxin exposures were higher during the middle decades of the 20th century than they are now (Solomon and Weiss, 2002; EPA, 2002) and that body burdens in older individuals are currently significantly higher than in younger individuals. However, the general time trend in many countries seems to be toward a slight decrease of dioxin levels in BM over past decade (LaKind et al. 2001). In some countries, the decrease has been quite dramatic, with levels reduced by as much as 50% (Dewailly et al. 1994). Coordinated WHO studies in Europe from 1986 to 1993 showed an average decrease in dioxin levels of approximately 35%, with consistently higher levels in industrial areas (Solomon and Weiss, 2002; IARC 1997).

Interestingly, dietary exposure makes up more than 90% of human dioxin intake. Schecter and colleagues (Schecter et al. 1994) measured levels of dioxins in U.S. food. The average daily food intake for adult was 0.3–3 pg toxic equivalent (TEQ)/kg body weight (bw). A nursing infant may consume an average of 35–53 pg TEQ/kg bw/day during the first year of life. Daily dioxin TEQ intake in boy from 1 to 4 years of age range from 1.4 to 32 pg/kg bw. Also vegetarian mothers have been shown to have lower levels of dioxin in their milk compared to women who eat a diet rich in meat (Somogyi and Beck, 1993). Finally, lactation is one of the main routes of dioxin excretion (Massart et al. 2005).

## Semivolatile Organohalogens

### Polybrominated biphenyls, polychlorinated biphenyls, polychlorinated dibenzo-p-dioxin and polychlorinated dibenzofurans

Polychlorinated biphenyls (PCBs) were widely used as flame retardants, in surface coatings and in electrical equipment such as transformers, and persistent PCBs occur as environmental contaminants around the world (Massart and Meucci, 2007). The PCBs belong to a large organohalogen chemical family which includes organochlorine insecticides, polychlorinated dibenzo-p-dioxins (PCDDs), polychlorinated dibenzofurans (PCDFs), polybrominated biphenyls (PBBs), PCBs, polybrominated diphenyl ethers (PBDEs). The PCDDs, PCDFs and certain PCB congeners represent classes of many halogenated aromatic compounds that exhibit similar mechanisms of toxic actions. There are 75 congeners of PCDDs, 135 congeners of PCDFs and 209 PCB congeners (Needham and Wang, 2002). All of these congeners could potentially be widespread and persistent environmental pollutants. The highly lipophilic and hydrophobic PCBs, dioxins (i.e. PCDDs and PCDFs), and related compounds tend to partition into soil and sediment, to bioconcentrate from water to acquatic animal, and to biomagnify up the multistep food chain (Massart et al. 2006b).

Humans are on the food chain top, eating meat and dairy products form herbivores, as well as fish and plants. However, not all of the congeners bioaccumulate in the food chain and hence are not found in the fatty stores in humans. For example, only 7 PCDDs and 10 PCDFs (but the most toxic members) are generally stored in human fatty tissues (Needham and Wang, 2002). Because of their dioxin-like activity, 12 PCBs are generally reported along with PCDDs and PCDFs. Therefore, approximately 30 different chemicals are reported as dioxin-like chemicals in humans.

The PCB concentrations in BM or in cord blood are dependent to the maternal PCB and dioxin body burden (Massart et al. 2005). This body burden is the result of PCB and dioxin-like accumulation over many years, especially in fat tissue, combined with low metabolic degradation and excretion rate (Massart et al. 2005; Webster and Commoner, 1994). As a result, the half-life is very long for higher chlorinated PCDD and PCDFs, ranging between 4 and 12 years, and for PCBs, ranging between 5 and 15 years (Flesch-Janys et al. 1996; Wolff et al. 1992).

Most of PCB monitoring studies were performed in Europe (Noren and Meironyte, 2000; Jensen and Slorach, 1991; Schoula et al. 1996; Polder et al. 1998; Dewailly et al. 1994; Cerna et al. 2007; Wilhelm et al. 2007). Because of no standard measuring protocols, it is difficult to assess BM trends of PCB contaminants. However, some researchers have speculated that, over the last 25 years, levels may have decreased slightly (Longnecker et al. 1997). In Sweden, Czech Republic and Germany, where data have been collected following fairly consistent methods over time, evidence of a downward trend has emerged (Noren and Meironyte, 2000; Cerna et al. 2007; Wilhelm et al. 2007).

Other studies evaluating PCB levels in women, have found BM concentrations were 4–10 times higher than in blood (Longnecker et al. 1997). For example, Patandin et al. (Patandin et al. 1999) reported a mean daily intake of 112–118 pg TEQ/kg bw in breast-fed infants and 6.3–6.5 pg TEQ/kg bw in 1- to 5-year-old children. Maternal age is positively related, and the period of previous breast-feeding is negatively related to maternal PCB and dioxin-like concentrations (Albers et al. 1996; Massart et al. 2005).

Because more than 90% of the total daily human exposure to PCBs and dioxin-like chemicals is made up of oral intake from food, whereas other routes (e.g. water, air and soil) contribute less than 10% of total exposure (Furst et al. 1992; Theelen et al. 1993), the role of diet has been investigated regarding BM pollution. In the U.S., fish consumption in the Great Lakes area has been associated with higher body burden of PCBs (Falk et al. 1999). In Canada, Inuit and fishing populations have higher PCB levels in BM than do urban population (Hirakawa et al. 1995). Fish and marine mammal consumption are not the only dietary exposures of concern. In the Czech Republic, the use of PCB-containing paint in grain silos led to BM levels higher than those found in neighboring regions and countries (Schoula et al. 1996; Cerna et al. 2007). Furthermore, short-term dietary regimens with low doses of dioxins during the lactation period showed no reduction in BM dioxin concentrations (Pluim et al. 1994).

### Polybrominated diphenyl ethers

PBDEs are widely used flame retardants. They are added to plastic material in televisions and computers, and are also found in construction materials, furniture and textiles (Meironyte et al. 1999). Unlike the PCBs and many of the organochlorine pesticides, the PBDEs are still widely used throughout the world. The production and use of PBDEs have steadily increased since the 1970s. PBDEs can enter the environment during the production and disposal of materials containing PBDE flame retardants, as well as during the lifetime of PBDE-containing products (Massart and Meucci, 2007). PBDEs are not chemically bound to plastics, so they can evaporate into indoor air or the outdoor environment (Hooper and McDonald, 2000). Once released, PBDEs can build up in environment and in living organisms, binding strongly to sediment and building up in fish and other aquatic organisms (Meironyte et al. 1999).

The PBDE similarity than dioxins and PCBs has been concern because their negative effects on health may prove to be similar, affecting hormone function and brain development (Darnerud et al. 2001; Massart et al. 2006a). Actually, there is worldwide interest to measure current PBDE levels in BM. In a Sweden study, archived samples collected between 1972 to 1997 showed logarithmic increase in the PBDE quantity detected in BM (Noren and Meironyte, 2000; Meironyte et al. 1999). Similar increasing trends of BM contamination were recently reported in remote populations living in Atlantic and Pacific areas of the U.S. and Canada (Fangstrom et al. 2005; She et al. 2007a–b). Although lower than North America data (Ryan et al. 2002; Schecter et al. 2003), PBDE values from Australia (Toms et al. 2007) were equal to or higher than those in Europe (Tsydenova et al. 2007; Jaraczewska et al. 2006; Fürst P 2006; Strandman et al. 2000; Kalantzi et al. 2004) and in Asia (Sudaryanto et al. 2005a; Bi et al. 2006; Eslami et al. 2006).

Actually, no restrictions have been placed on the production and use of PBDEs, but some European governments has announced an intention to ban PBDEs in products sold, based partly on BM detection of these chemicals in BM (Noren and Meironyte, 2000; Darnerud et al. 2001).

## Dichlorodiphenyltrichloroethane

Dichlorodiphenyltrichloroethane (DDT) is a commercial organochlorine insecticide that has been widely used for agricultural purposes, as well as in public health programs to eradicate malaria (Lopez-Carrillo et al. 1996). The DDT half-life is approximately 4 yrs while DDE, the DDT’s major metabolite, has 6-yrs-half-life (Noren and Meironyte, 2000). Therefore, DDT and its byproducts can persist in soil and sediments for more than 15 years, and are known to bioaccumulate in animal tissues (Massart et al. 2005). Ulrich and colleagues (Ulrich et al. 2000) reported that concentrations of organochlorine pesticides in different unexposed human populations were generally only 2.7–120 times lower than *o,p*′-DDT concentrations found cause hormone effects in mice. However, in Israel, *o,p*′-DDT blood concentrations were found to be as high as 32 ng/mL (Toppari et al. 1996), nearly double the minimal estrogenic blood level of 18 ng/mL observed in *in-vivo* mice studies (Ulrich et al. 2000). However, because DDE and DDT are attracted to fat, their BM levels are often six to seven times higher in mother’s milk than in her blood (Massart et al. 2005).

The primary DDT/DDE source of human exposure is through the food chain: DDT and DDE are stored in fat of fish, dairy and meat products (Lopez-Carrillo et al. 1996; Hovinga et al. 1993). In general, DDT-contamination is parallel to environmental PBC-pollution. Fish obtain organochlorines from sediments of fresh water bodies; for example, high levels of DDT and DDE has been repeatedly measured in fish caught in the Great Lakes (Hovinga et al. 1993). Also, beef, poultry, eggs and dairy have been environmentally exposed to DDT, DDE and PCBs (Solomon and Weiss, 2002). In a German population-based study, serum DDT and PCB levels were positively associated with between beef, lamb and saltwater fish consumption (DeVoto et al. 1998). Organichlorine residues have also been detected in fruits, vegetables and grains (Stehr-Green et al. 1988), though food levels vary substantially depending on the food source. Home-grown produce from residences with high soil contents of DDT and PCBs could have higher levels of these compounds than store-bought fruits and vegetables (Laden et al. 1999).

As of 1995, DDT have been banned for all uses in 49 countries and restricted to vector control in 23 (PANNA, 1995). The average levels of DDT in BM have varied considerably among nations. After the restriction and the ban of DDT in some nations (in mid-1970s), average BM levels decreased substantially in many (Noren and Meironyte, 2000; Jensen and Slorach, 1991; Fürst et al. 1994; Wickstrom et al. 1983; Schade and Heinzow, 1998; Chao et al. 2006; Shen et al. 2007; Jaraczewska et al. 2006) but not in all (Kunisue et al. 2006; Subramanian et al. 2007). Furthermore, several Asian studies (Sudaryanto et al. 2006; Kunisue et al. 2004a; Kunisue et al. 2004b; Minh et al. 2004; Sudaryanto et al. 2005b; Yu et al. 2003) reported accumulated DDT/DDE in BM close to or even higher than the tolerable daily intake guidelines proposed by Health Canada (Van Oostdam et al. 1999).

## Heavy Metals

A number of potentially toxic metals such as lead, mercury and cadmium have been reported in BM monitoring studies, and their mean values in human milk vary in a wide range. Indeed, mean lead concentrations ranged from 5 to 277 mg/ml until 1973 (Dillon et al. 1974) and then, Iyengar and Woittiez (1988) suggested a BM reference value of 30 μg/l. Souad et al. (2006) recently reported a BM lead content of 3.1–117.4 μg/l in Moroccan women, while Al-Saleh et al. (2003) found 31.67 μg/l lead in Saudi Arabia. In a Turkish study, BM values of lead and cadmium were 14.6 μg/l and 2.8 μg/l, respectively (Turan et al. 2001). On the other hand, cadmium (0.14–0.19 μg/l) and lead (0.15–0.48 μg/l) ranges recently reported in Greek population (Leotsinidis et al. 2005) were among the lowest reported in the literature. Worldwise mercury contaminations ranging from 0.03 ng/ml in Canada (Winfield et al. 1994) to 200 ng/ml in Iraq (Bakir et al. 1973), were systematically reviewed by Dorea (2004).

This wide variation shows a strong dependence of heavy metal content on various factors including the local environment, socioeconomic conditions of the family and local diet and habits. For example, plant workers showed the higher presence of heavy metals (e.g. lead, mercury and manganese) in their BM and blood samples compared to the residents of the area and the subjects living outside the industrial environment, respectively (Sharma and Pervez, 2005). Leotsinidis et al. (2005) showed that the mother’s place of residence plays a significant role in BM lead content: mothers living in urban areas had higher lead concentration in BM (*p* < 0.001) compared to those living in rural areas. This difference is most likely due to the higher traffic density within the cities. The majority of previous studies supported the view that women living in the urban areas with heavy road traffic and industrial activity have BM lead concentrations significantly higher than women leaving in rural areas (Huat et al. 1983; Guidi et al. 1992; Frkovic et al. 1997; Saleh et al. 1996). However, airborne particles are the main source of maternal exposure, they are not direct modulators of BM lead (Dorea, 2004).

In mature milk, positive associations between BM mercury and fish consumption (Oskarsson et al. 1995; Oskarsson et al. 1996) and between BM mercury and amalgam fillings (Oskarsson et al. 1995; Oskarsson et al. 1996; Drasch et al. 1998; Vimy et al. 1997) have been demonstrated. However, in transitional milk, such associations were not statistically significant (Klemann et al. 1990). On the other hand, cadmium levels in BM are significantly associated with cigarette smoking (Hallen et al. 1995). One Germany study showed a direct relationship between the number of cigarettes a mother smokes per day and BM cadmium level (Radisch et al. 1987). It is interesting to note that some studies indicate that infant’s exposure to cadmium from soy formula is about 20-fold higher than BM levels generally found (Oskarsson et al. 1998).

Unlike other persistent organic pollutants, heavy metals (i.e. lead, mercury and cadmium) appear in human milk at smaller concentrations than lipid-soluble chemicals and are about 20% of the level found in blood from the same person (Golding, 1997). This is attributed to their low lipid-solubility and high binding to erythrocytes. As a result, infants are likely to be exposed to higher levels before birth than during breastfeeding (Needham and Wang, 2002). Nonetheless, toxic metals in BM are important as additional pathway of exposure and as indicator of likely prenatal exposures (Solomon and Weiss, 2002; Cerna et al. 2007).

## Volatile Organic Compounds

In a European collected analysis by WHO, 26 halogenated hydrocarbons, 17 aldehydes, 20 ketones, 11 alcohols, 2 acids, 3 ethers, 1 epoxide, 14 furans, 26 other oxygenated compounds, 4 sulfur-containing compounds, 7 nitrogen-containing compounds, 13 alkanes, 12 alkenes, 7 alkynes, 11 cyclic hydrocarbons and 15 aromatic compounds were found including significant amounts of hexanal, limonene, and dichlorobenzene (WHO, 1996). As in most cases, the “other chemical” category contains a variety of chemicals ranging from contemporary pesticides, polycyclic aromatic hydrocarbons, nicotine, ethanol, phthalates, musk xylenes and phytoestrogens such as genistein (Needham and Wang, 2002).

Numerous organic solvents have been detected in BM, including benzene, chloroform, methylene chlorine, styrene, perchloroethylene, toluene, trichloroethylene, 1,1,1-trichloroethane and xylene (Labreche and Goldberg, 1997). Solvents included many chemical classes with varying properties, defined more by their use than their chemistry or toxicity. These chemicals were present in paints, varnishes, thinners, dry-clearing fluids, some glues, degreasers and gasoline (Solomon and Weiss, 2002). In general, organic solvents are highly volatile and readily absorbed through the skin. They were also common water contaminants. As result of their widespread presence in the environment, solvents were found in human urine, exhaled breath, blood, and fat (Lordo et al. 1996). Because solvents are relatively short-lived in the body, detection implied recent exposure.

BM levels of these chemical compounds may be higher than blood levels in part because breast tissue does not eliminate solvents as quickly as does blood (Labreche and Goldberg, 1997). Perchloroethylene, in particular, is known to concentrate in BM about 3-fold higher than its blood levels (Fisher et al. 1997). Due to limited data, it is possible only to conclude that some solvents get into BM; no information relevant to the levels of exposure, geographic differences or time trends are available.

## Conclusions

In the last decades, the continued efforts of scientists to measure environmental pollutants in human milk is important for defining the true toxic contribution of these chemicals to public health, especially to the infant health. However, it also illuminates there are several gaps in current knowledge including: (a) insufficient information on the nature and levels of contaminants in BM, (b) lack of consistent protocols for collecting and analyzing BM samples, (c) lack of toxicokinetic data, and (d) lack of data on health outcomes that may be produced in infants by exposure to chemicals in BM. These gaps in information impede risk assessment and make difficult the formulation of evidence-based health guidance. To address these issues, there is a need for a carefully planned and conducted national BM monitoring effort both in industrially developed as well as in developing countries. Additionally, to assess health outcomes of toxic exposures via BM, it will be necessary to examine children prospectively over many years in longitudinal epidemiologic studies that use standardized examination protocols that specifically assess BM exposures. Finally, current risk assessment methods need to be expanded to include consideration of the potential risks posed to infants and children by exposure to chemical residues in BM (Landrigan et al. 2002).

Excluding specific poisoning situations, present data do not support to altering WHO’s recommendation for exclusive breastfeeding for 6 months as a global public health recommendation and the provision of safe and appropriate complementary foods, with continued breastfeeding for up to 2 years of age or beyond (WHO, 2002). Risk management should thus aim to limit intake of contaminated food by the mother rather than restrict breastfeeding.

## Abbreviations

AAP: American Academy of Pediatrics
BW: body weight
BM: breast milk
DDE: dichlorodiphenyl-chloroethane
DDT: dichlorodiphenyltrichloroethane
HCB: hexachlorobenzene
HCH: hexachlorocyclohexane
HHS: Department of Health and Human Services
NOAEL: no-observed-adverse-effect level
PBB: polybrominated biphenyl
PBDE: polybrominated diphenyl ether
PCB: polychlorinated biphenyl
PCDD: polychlorinated dibenzo-p-dioxin
PCDF: polychlorinated dibenzofuran
TCDD: 2,3,7,8-tetrachlorodibenzo-p-dioxin
TEQ: toxic equivalent
WHO: World Health Organization
